# Interpersonal capitalization in esports enhances players’ psychological resources and well-being

**DOI:** 10.1038/s41598-025-17968-1

**Published:** 2025-09-25

**Authors:** Lukasz D. Kaczmarek, Dariusz Drążkowski

**Affiliations:** 1 Adam Mickiewicz University, Poznań,, Poland; 2 Faculty of Psychology and Cognitive Science, Adam Mickiewicz University, 89 Szamarzewskiego Street, Poznań, 60-658 Poland

**Keywords:** Psychology, Human behaviour

## Abstract

Interpersonal capitalization refers to psychosocial benefits derived from communicating personal accomplishments to others and receiving active-constructive (enthusiastic) feedback. Little is known about the extent to which interpersonal capitalization generalizes to the digital environment of esports. In esports, players compete through video games, seeking accomplishments and often communicating with other players. Building upon interpersonal capitalization theory, we hypothesized that players who more frequently communicate their accomplishments to others and perceive more active-constructive responses would experience enhanced psychological resources (self-esteem, self-efficacy, and optimism), thereby improving both hedonic and eudaimonic well-being. In contrast, perceived active-destructive feedback (e.g., demeaning feedback pointing out the downsides of the accomplishment) would be associated with lower psychological resources and well-being. Esports players (*N* = 291, 50.2% male) completed a two-wave online survey with measurements four weeks apart. We found that players who perceived more active-constructive feedback were more likely to increase optimism (direct effect) and positive emotions (indirectly via increased optimism) over the next month. Perceived active-destructive feedback predicted decreases in self-esteem, and players with lower self-esteem were more likely to exhibit more gaming addiction symptomatology. These findings are the first to present specific pathways through which esports players benefit from shared accomplishments and feedback. It highlights the viability of interpersonal capitalization in digital social environments.

**Supplementary Information:**

The online version contains supplementary material available at 10.1038/s41598-025-17968-1.

## Introduction

Esports (electronic sports) refers to competitive video gaming where individuals or teams compete against each other in multiplayer video games^1–3^. At a professional level, esports typically involve organized tournaments. Nevertheless, most esports players are hobbyist gamers who participate recreationally, engaging primarily as a leisure activity. Popular esports titles include *League of Legends*,* Counter-Strike: Global Offensive*, and *FIFA.*

Esports players frequently seek peer validation by communicating their accomplishments and expecting feedback from other players^4^. We focused on interpersonal capitalization, a theoretical framework that presents the benefits individuals derive from communicating personal achievements and receiving active-constructive (enthusiastic) feedback^5–11^. The psychological benefits of interpersonal capitalization include increases in psychological resources and well-being. These benefits have been documented across several life domains^10^. However, the benefits of interpersonal capitalization for esports remain unexamined, even though esports provide regular opportunities for communicating achievements and social interaction for many players daily. Thus, building upon the interpersonal capitalization theory^10^, we aimed to examine the associations between capitalization and esports players’ psychological resources and well-being.

To our knowledge, this study is the first to focus on interpersonal capitalization in esports. While studying this population, it is imperative to focus not only on the benefits (e.g., positive emotions) but also on mental health risk factors, such as gaming addiction. Moreover, psychological resources (e.g., self-efficacy) are likely to explain the mediating mechanism between interpersonal capitalization and psychological outcomes. Finally, longitudinal designs are needed to establish effects that evolve in players.

## Interpersonal capitalization

When individuals experience positive events, they derive psychosocial benefits by communicating these successes to others and receiving constructive feedback, a process known as capitalization^5,10,11^. Capitalization complements the social support and coping perspective, which primarily focuses on how individuals seek assistance from others to mitigate stress or recover from failures^11,12^. Unlike coping, which addresses negative events to minimize their detrimental effects, capitalization enhances positive experiences by amplifying their benefits^10^. Importantly, the effects of capitalization are distinct from the benefits associated with sharing negative experiences, which involve social support efforts in coping with negative events^13^. Finally, the interpersonal capitalization framework emphasizes not only the role of received feedback but also the effects of merely expressing positive events.

Capitalization theory suggests that fully experiencing benefits requires individuals both to share their positive events (capitalization attempts) and to perceive and evaluate responses from others (perceived responsiveness). However, empirical evidence consistently indicates that simply communicating positive events to others can itself yield psychological and relational benefits, irrespective of the nature or quality of responses received^5,11^. Daily diary studies show that individuals report increased positive affect and enhanced life satisfaction on days when they share positive experiences, even after controlling for the intrinsic positivity of the events themselves^5,11,13^. Proposed mechanisms for these benefits include emotional amplification or savoring of the event while communicating and cognitive elaboration required for communicating which can increase the event’s memorability^5,14^.

Additional psychological benefits derived from such positive self-disclosures are contingent upon the nature of the response provided by their conversation partners. There are four ways to react when a person makes a capitalization attempt^5^: active-constructive (e.g., “Congratulations! Tell me more!“) passive-constructive (e.g., “Not bad”), passive-destructive (e.g., “Watch how I will play now”), and active-destructive (e.g., “You could have tried harder”). Research has shown that only active-constructive responses correlate positively with healthy relationship outcomes, whereas passive and destructive responses correlate negatively^5,14^. This suggests that the lack of active support often functionally equals a destructive response in its effects, as passive support might feel insufficient and fail to provide emotional validation^5,12,15,16^. Active-destructive responses consistently produce the most adverse immediate relational consequences, particularly severe emotional harm, increased relational conflict, and reduction in perceived responsiveness. These responses actively undermine the primary purpose of capitalization (validating and amplifying positive experiences), leading directly to negative relational trajectories. Studies emphasized that active-destructive responses clearly and unambiguously signal relational rejection, criticism, or lack of emotional support, strongly predicting reduced satisfaction and intimacy.

Active-constructive feedback is described as enthusiastic and elaborative and is considered healthy and effective because when conversation partners respond enthusiastically, individuals sharing their successes can maximize their benefits^12,17^. Receiving enthusiastic feedback conveys validation of the emotional experience, i.e., the person who communicated the event is more convinced that there were good reasons to be happy in the first place. In contrast, active-destructive feedback (e.g., focusing on the bad aspects of good events) undermines the certainty that the event can be considered positive, leading to suppressed positive emotions. Second, active-constructive feedback can provide further emotional amplification and savoring in an interpersonal interaction, leading to more positive emotions and satisfaction^18^. Moreover, after receiving active-constructive feedback, the sharer increases the belief that their peers are responsive to their emotional needs, which leads to more social comfort and less distress^13,19,20^. Finally, active-constructive feedback fulfills essential psychological needs for belonging, competence, and esteem, reinforcing positive self-concept^21^. In contrast, the other three types of feedback (destructive or passive) are characterized as unenthusiastic, inattentive, and undermining and are considered dysfunctional^5,9,11,22^ as they may undermine the need for autonomy and competence.

Thus, the processes studied within the interpersonal capitalization framework can also contribute to the Cognitive Evaluation Theory, which posits that feedback influence intrinsic motivation by affecting the psychological needs for competence and autonomy^23^. CET distinguishes between informational feedback, which supports competence and enhances intrinsic motivation, and controlling feedback, which undermines autonomy and reduces intrinsic motivation. Active-constructive feedback aligns closely with CET’s concept of informational feedback as it validates the individual’s experience and enhances their sense of competence. This type of feedback also supports autonomy by allowing individuals to feel freely acknowledged and emotionally supported in their achievements. In contrast, active-destructive feedback mirrors controlling feedback in CET by undermining autonomy and competence. Such feedback can diminish intrinsic motivation by signaling relational rejection and invalidating the individual’s positive experience. Thus, the types of feedback identified in interpersonal capitalization theory can be understood through the lens of CET as social responses that differentially satisfy or frustrate the needs for autonomy and competence, ultimately influencing motivation, well-being, and relational quality.

Interpersonal capitalization has been studied in social settings, such as romantic relationships^10,17^. The competitiveness of esports and the opportunity to share achievements among players make it an adequate environment to study interpersonal capitalization in a novel context.

### Psychological resources in gaming

The abundance of resources helps individuals appraise stressors more positively, cope effectively with challenges, and remain resilient in the face of stress^24^. We focus on performance-related resources, such as self-efficacy^25–27^, self-esteem^28–30^,, and optimism^31–33^. A positive mindset is characterized by expecting favorable outcomes during adversity (optimism), believing that personal effort can overcome obstacles (self-efficacy), and maintaining a positive sense of self-worth (self-esteem). Consequently, well-being is closely linked to self-esteem^34^, optimism^35^, and self-efficacy^36^. The relationship between psychological resources and video gaming is complex and multifaceted. Gaming frequency has been positively linked to increased self-efficacy, optimism, and self-esteem^37,38^. Researchers suggested that some gaming environments may foster an optimistic motivational style^39^. Moreover, high game-related self-efficacy is associated with greater motivation to play and enhanced performance in esports^40–42^. In contrast, low self-esteem has been identified as a risk factor for gaming disorder^43^. Thus, while gaming can bolster psychological resources such as self-efficacy and optimism, it can also undermine well-being, mainly when these resources are scarce or depleted. Previous studies indicate the role of active-constructive feedback on self-esteem, optimism, and self-efficacy^6,9,44–47^. Thus, it is reasonable to expect that active-constructive capitalization can enhance psychological resources.

Another important distinction is between the stable levels of resources attributable to personality traits and more dynamic aspects that can vary across specific periods. This is an important distinction because many players aim to maximize their resources within a specific period, e.g., a gaming tournament. Also, casual players are likely to experience short-term variations in psychological resources contingent on their current experiences, including social feedback on their performance. Thus, it is important to control for the stable component that is unlikely to change while focusing on variation that can be attributable to external factors. This requires longitudinal designs that can control stability using autoregression and model the remaining variability to account for change. For instance, self-efficacy is relatively stable but can be increased by interventions^48^.

Moreover, we focus on domain-specific resources rather than general. For instance, we focus on self-esteem and self-efficacy as a gamer rather than a person in general across life domains, as these components can, to some extent, differ with the specific resources being less stable based on a more dynamic and flexible gaming environment than real-life circumstances. Moreover, we focus on game optimism rather than general optimism, as they can also differ.

## Well-being in esports

Well-being is a construct encompassing hedonic and eudaimonic perspectives^49^. The hedonic perspective is characterized by high positive and low negative emotions, and overall life satisfaction^50^. Emotions play a crucial role in video gaming, offering intense emotional experiences that range from the straightforward joy of winning a challenging game to the unexpected relief after losing^4,51–53^. Positive emotions bring tangible benefits such as better performance and teamwork^7,54^. In contrast, negative emotions have been linked to poorer performance^55,56^. The eudaimonic perspective is often conceptualized as flourishing^57,58^. Individuals who flourish lead a purposeful life, maintain supportive relationships, engage in meaningful activities, and contribute to the well-being of others. These eudaimonic facets are particularly relevant to esports, where the quality of social interactions and the sense of personal achievement can influence long-term success and well-being.

Previous research justifies why feedback in a single life domain, such as esports, can have long-term impacts on overall psychological well-being. Studies have established connections between engagement in specific life domains and general psychological well-being, particularly when those domains hold significant personal relevance for the individual^59,60^. Specifically, research within esports contexts highlights how social interactions during gaming elicit strong emotional and psychological responses, significantly affecting gamers’ daily experiences and long-term psychological states^4^. Furthermore, parallel findings from sports research - a related domain - demonstrate that domain-specific engagement, particularly in socially supportive environments like team sports, enhances self-esteem and overall psychological health^61^. These studies underscore the broader psychological benefits of active engagement in socially supportive, domain-specific interactions, reinforcing the relevance and potential long-term impact of positive feedback within the esports domain.

## Gaming addiction

While most gamers engage in video games in a controlled way, esports players are at a heightened risk of problematic gaming^62^. Gaming addiction presents a compulsive use of video games that negatively influences gamers’ lives^63^and undermines well-being by contributing to adverse life outcomes^64,65^. Players with deficits in psychological resources, such as low self-esteem^43^, are particularly prone to developing gaming addiction. Interpersonal capitalization may be associated with gaming addiction in several ways, e.g., active-constructive capitalization may enhance psychological resources, which could indirectly reduce gaming addiction.

## Present study

We aimed to examine the role of interpersonal capitalization within the digital context of esports. We focused on how the frequency of communicating positive events, as well as perceived positive and negative social feedback, affects players’ psychological resources and well-being. We focused on assessing active-constructive (positive) and active-destructive (negative) feedback, as these forms of feedback have been shown in previous research to exert the most pronounced effects on psychological outcomes^5,12,15^. Given the increasing prominence of esports as a leisure activity and the critical role of social interactions in gaming, it is essential to understand how those types of social feedback on performance influence players’ psychological outcomes. We defined social feedback as the verbal and non-verbal responses that individuals perceive from their peers when they share their accomplishments. This includes verbal expressions (e.g., congratulations or criticism) as well as non-verbal cues (e.g., emojis, in-game gestures). To examine changes in variable levels, we conducted a longitudinal study, building on prior research demonstrating the long-term positive effects of active-constructive capitalization^20,66^.

We hypothesized that capitalization attempts and perceived active-constructive feedback received by players after sharing their gaming accomplishments would be associated with higher levels of psychological resources such as self-esteem, self-efficacy, and optimism, which in turn would relate to greater hedonic well-being, reflected in higher levels of positive emotions, life satisfaction, and eudaimonic well-being, as well as lower levels of gaming addiction symptomatology. Conversely, we hypothesized that perceived active-destructive feedback would be associated with lower levels of these psychological resources, and with higher negative emotions and potentially maladaptive outcomes such as gaming addiction. We controlled for gender differences as they are often observed in esports^67–70^.

## Method

### Participants

The analyzed sample involved 291 participants (50.2% male and 49.8% female) aged between 18 and 40 years (*M* = 29.72, *SD*= 5.49) recruited through the Prolific research panel^71^. We recruited 360 participants (50% women and 50% men) at Time 1 (T1), and 296 completed the second measurement four weeks later (18% dropout). We removed five participants who failed attention checks or reported incongruent demographic data (e.g., differences in reported age). Participants were from the UK (64.3%) and the USA (35.7%). The inclusion criteria required participants to play esports, be fluent in English, and commit to completing both measurements. Regarding employment, 57.4% were full-time workers, 12.4% were unemployed, 9.3% were part-time workers, and 25.1% were students. Ethnically, 66% identified as White, 14.8% as Asian, 10% as Black, 7.6% as mixed, and 1.7% as other. Participants who dropped out were younger (*M* = 27.67, *SD* = 4.94) than those who completed both measurements (*M* = 29.80, *SD* = 5.56), *t* (358) = −2.77, *p* <.01, Cohen’s *d* = − 0.39, indicating a small-to-medium effect size. Participants who dropped out were also more likely to communicate good in-game events to other players (capitalization attempts) (*M* = 4.77, *SD* = 1.54) than those who completed both measurements (*M* = 4.30, *SD* = 1.54), *t* (358) = 2.17, *p* <.05, Cohen’s *d* = 0.31, indicating a small difference between groups. All other differences were non-significant, *p* >.05.

Participants reported playing the following games most frequently (up to three choices per participant): Call of Duty (40.9%), FIFA (35.1%), League of Legends (32.0%), Fortnite (28.5%), Overwatch (18.9%), Rocket League (11%), and Valorant (11%). The majority identified as recreational players (96.9%), and some were professional players (2.1%) or identified as neither (1%). Their esports experience ranged from 1 to 30 years (*M* = 9.87, *SD* = 5.54). We asked participants about their daily gaming time over the past 30 days with the question, “In the last 30 days, how much time have you spent on esports games each day? Please provide the number in hours.” However, many participants provided total monthly hours (e.g., 100 h) instead of daily averages, making the data not useful for analysis. Participants played on PCs (90.4%), video game consoles (84.2%), and handheld consoles (67.7%).

Participants received $1.5 after completing each session. The Research Ethics Committee of the Faculty of Psychology and Cognitive Science approved the study and the methods (No: 4/05/2022). Informed consent was obtained from all participants.

## Procedure

Upon receiving the invitation through the Prolific platform, participants accessed an online questionnaire hosted on Qualtrics. They answered questions confirming the eligibility criteria and completed the full set of study questionnaires. At the end of the questionnaire, participants were informed that they would receive an invitation to the second part of the study in four weeks. Four weeks later, they were sent the same questions and thanked for their participation upon completion.

## Measures

*Overview.*Recent psychometric works indicate that shorter scales have similar psychometric properties and yield similar results to extended versions^72–75^. Thus, briefer scales might be preferred in online studies where the patience and reliability of participants tend to drop for more extended measurements^76^. When briefer scales were unavailable, we selected items with the highest loadings from more extended versions based on data from a preliminary study that used longer scales. All items from the shortened scales are presented in Table 1 (online supplement). We estimated the scales’ reliability by calculating McDonald’s omega, which is increasingly recommended as an alternative to the widely used Cronbach’s alpha^77^. Omega is a more general measure of internal consistency because, unlike alpha, it does not assume equal item contributions. Thus, omega typically provides a more accurate reliability estimate than alpha.

*Perceived Responses to Capitalization Attempts in Gaming.*We measured the frequency with which players received specific feedback from other players when communicating positive in-game experiences. We used a modified version of Perceived Responses to Capitalization Attempts, which includes items that assess both perceived active-constructive and active-destructive responses^5^. We used three items for perceived active-constructive feedback: “Other players usually react to my good fortune enthusiastically,” “I sometimes get the sense that other players are even more happy and excited than I am,” “Other players often ask a lot of questions and show genuine concern about the good event.“). We also used three items for perceived active-destructive feedback: “Other players often find a problem with it,” “Other players remind me that most good things have their bad aspects as well,” and “Other players point out the potential downsides of the good event.” This was measured using a scale from 0 to 6 (0 = never, 6 = always). The scales’ reliability was acceptable for active-constructive feedback, ω = 0.70 (T1) and ω = 0.73 (T2), and active-destructive feedback, ω = 0.88 (T1) and ω = 0.84 (T2).

*Perceived Frequency of Capitalization Attempts in Gaming.* We asked participants to estimate how often they shared their accomplishments with other players. “How often do you communicate good in-game events (e.g., an important win, significant progress) to players you keep in touch with within the game (e.g., your teammates or your friends).” Participants responded on a scale from 0 to 6 (0 = never, 6 = always). The Pearson correlation coefficient between the two administrations was *r* =.59, *p* <.01, indicating moderate test-retest reliability.

*Self-Efficacy.* We measured players’ belief in their ability to handle challenges during gameplay with three revised items from the Self-Efficacy Questionnaire^78^: “When I am confronted with a problem in a game, I can usually find several solutions,” “If I am in trouble in the game, I can usually think of a solution,” “I can usually handle whatever comes my way in the game.” Participants rated their agreement with statements on a 7-point Likert scale (1 = strongly disagree, 7 = strongly agree). The scales’ reliability was high, ω = 0.86 (T1) and ω = 0.87 (T2).

*Self-Esteem.* We measured participants’ self-satisfaction and perceived self-worth as a player with three revised items from the Self-Esteem Scale^29^: “I feel that I have a lot of good qualities as a player,” “I have a positive attitude about myself as a player,” “On the whole, I am satisfied with myself as a player.” Participants rated their agreement on a 7-point Likert scale (1 = strongly disagree, 7 = strongly agree). The scales’ reliability was high, ω = 0.87 (T1) and ω = 0.89 (T2).

*Optimism.* We measured participants’ favorable expectations regarding the future using three revised items from the Life Orientation Test^79^: “I’m always optimistic about my future during the game,” “During the game, I rarely expect good things to happen to me” (reverse-scored), “Overall, I expect more good things to happen to me during the game than bad.” They rated their agreement with several statements on a 7-point Likert scale (1 = strongly disagree, 7 = strongly agree). Of the scales used, this scale included a reverse-scored item, which could influence its reliability. The reliability was acceptable for the first measurement, ω = 0.72 (T1), but somewhat lower for the second measurement, ω = 0.62 (T2).

*Positive and negative emotions*. Participants reported the frequency of experiencing six positive emotions (e.g., happy, joyful, content) and six negative emotions (e.g., angry, sad, afraid) on a 5-point Likert scale (1 = very rarely or never, 5 = very often or always)^57^. Participants were asked to indicate how well each adjective reflected their feelings over the past four weeks. The scales’ reliability was high for positive emotions, ω = 0.92 (T1) and ω = 0.92 (T2), and negative emotions, ω = 0.88 (T1) and ω = 0.89 (T2).

*Satisfaction with life.* We measured how satisfied participants were with their lives using an abbreviated three-item version of the *Satisfaction with Life Scale*^72,80^: “I am satisfied with my life,” “In most ways my life is close to my ideal.“, “The conditions of my life are excellent.” Participants rated their agreement with statements on a 7-point Likert scale (1 = strongly disagree, 7 = strongly agree). The scale reliability was very high, ω = 0.95 (T1) and ω = 0.95 (T2).

*Eudaimonic Well-Being.*We measured eudaimonic well-being with the Flourishing Scale^57^. Participants responded to three items: “I lead a life that has meaning and purpose”, “I am a good person and live a good life,” “I am engaged and interested in my daily activities.” Participants rated their agreement with various statements on a 7-point Likert scale (1 = strongly disagree, 7 = strongly agree). The reliability was high, ω = 0.90 (T1) and ω = 0.92 (T2).

*Video Game Addiction.*We measured whether participants experienced symptoms of gaming addiction through the Gaming Disorder Test^63^. This scale was developed according to WHO guidelines and the ICD-11 classification. It includes four items addressing the key diagnostic criteria for a gaming disorder, such as “I have had difficulties controlling my gaming activity,” “I have given increasing priority to gaming over other life interests and daily activities,” “I have continued gaming despite the occurrence of negative consequences,” “I have experienced significant problems in life (e.g., personal, family, social, education, occupational) due to the severity of my gaming behavior.” We used the complete scale. Participants responded using a scale from 1 (“Never”) to 5 (“Very often”). The scale’s reliability was high, ω = 0.85 (T1) and ω = 0.90 (T2).

### Analytical strategy

We used Path Analysis with Mplus 8.5^81^ to examine the relationships between interpersonal capitalization (active-constructive and active-destructive responses) and gamer’s psychological resources (self-esteem, self-efficacy, and optimism) and, in turn, well-being (positive emotions, negative emotions, satisfaction with life, flourishing, and gaming addiction). We controlled for age and gender.

We built a two-wave model, which supports the assumption of temporal separation between predictors and outcome variables^82^, essential for testing change and mediating effects^83^. We tested how predictors at Time 1 (T1) are related to outcomes at Time 2 (T2) while controlling for the baseline (T1) levels. Thus, due to the control of auto-regressive effects, the regression coefficients reflected how much the predictors contributed to changes in the outcomes at T2 beyond what is explained by their initial outcome levels at T1. This approach isolates the associations between predictors and changes in outcomes, providing a clearer understanding of how these variables relate over time. For indirect effects, we tested the products of two path coefficients: from X at Time 1 to M at Time 2 multiplied by the path leading from M at Time 1 to Y at Time 2^82^. To explore whether age moderated the associations between interpersonal capitalization and psychological resources, we introduced interaction terms between age and each interpersonal capitalization variable at Time 1. These interaction terms were computed by multiplying the centered age variable by the respective predictors.

We accounted for effect sizes by interpreting standardized beta sizes that present how many standard deviations a dependent variable will change per standard deviation increase in the predictor variable. Standardized beta coefficients of 0.10, 0.30, and 0.50 represent small, medium, and large effect sizes^84^.

We used the robust Maximum Likelihood Estimator (MLR) to evaluate the fit of the path model^81^. MLR scales the data to account for possible deviations from normality and heteroscedasticity. We evaluated the model fit based on the Root Mean Square Error of Approximation (RMSEA), the Comparative Fit Index (CFI), and the Standardized Root Mean Square Residual (SRMR). The RMSEA values below 0.06 indicate a good fit, while values up to 0.08 are acceptable, especially for more complex models^85–87^. The CFI, which compares the fit of a target model to a baseline model, is deemed acceptable at values above 0.90 and good at values exceeding 0.95. SRMR < 0.08 is considered acceptable.

## Results

**Fig. 1:**
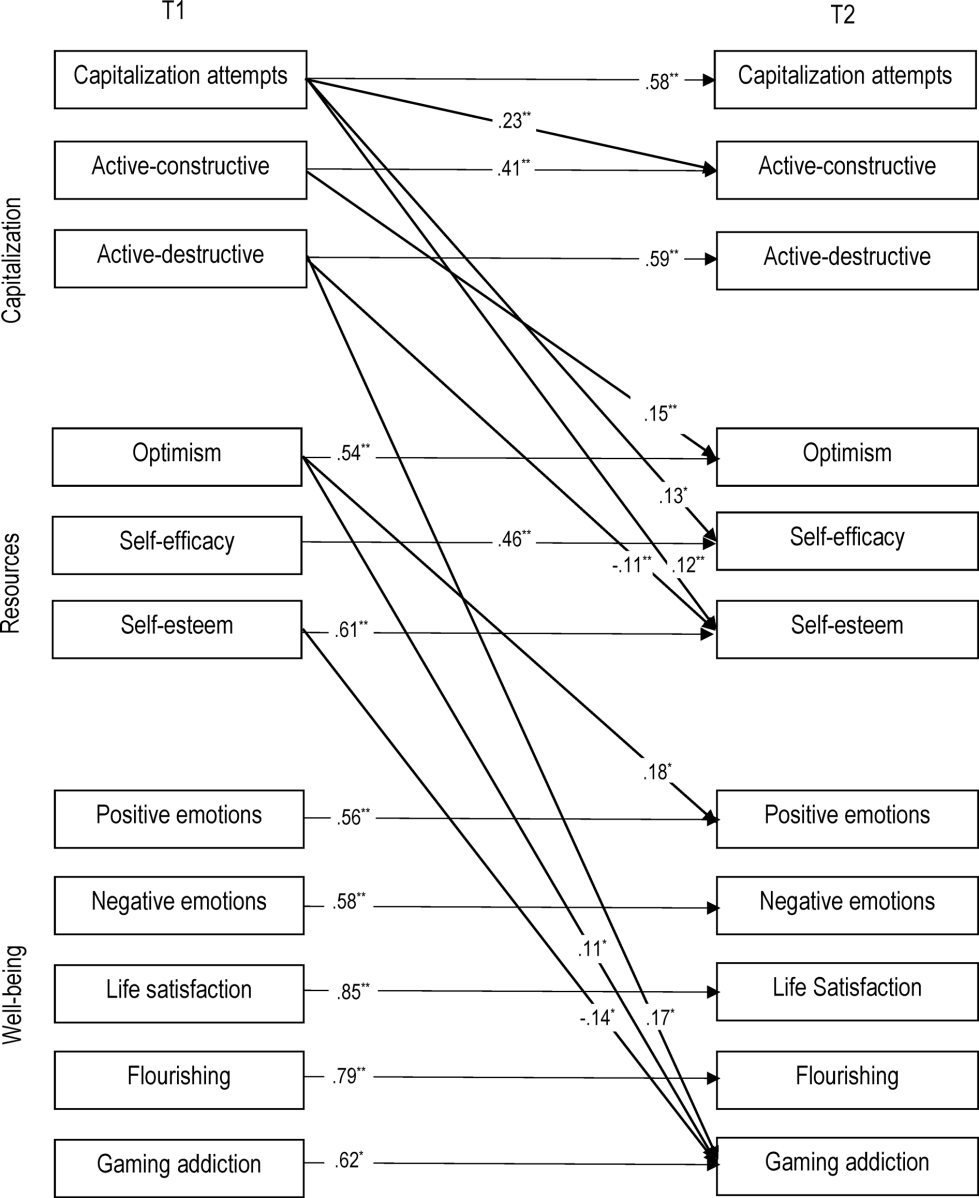
Path model of interpersonal capitalization, gaming psychological resources, and well-being. *Note.* Gender and age were controlled but are not shown for clarity; details are provided in the text. T1 = first measurement, T2 = measurement four weeks later. Thinner lines represent autoregressions. Insignificant paths are omitted for clarity. ^*^p < .05, ^**^p < .01, ^***^p< .001.

 Descriptive statistics and correlations between the study variables are presented in Table 1 (online supplement). The model is in Fig. 1. The summary of the direct effects is presented in Table 2 (online supplement) and the indirect effects in Table 3 (online supplement). The model fit the empirical data adequately, χ²(83) = 193.79, *p* <.01, RMSEA = 0.067, CFI = 0.953, and SRMR = 0.086.

We found that players who made more capitalization attempts, regardless of the type of feedback, were more likely to show improvements in self-esteem and self-efficacy over time. These increases reflected small effect sizes. Players who made more capitalization attempts were more likely to perceive more active-constructive responses next month, with small-to-medium effect sizes.

Moreover, participants who perceived more active-constructive responses from other players were more likely to report slight increases in optimism the following month. Furthermore, there was an indirect effect between receiving active-constructive responses and positive emotions mediated by optimism, b = 0.015, *SE* = 0.007, *p* =.029. Specifically, players who received more active-constructive responses experienced greater positive emotions due to their increased optimism about their game performance.

As capitalization attempts predicted increases in perceived active-constructive responses, there was also an indirect effect of capitalization attempts on optimism via perceived active-constructive responses, b = 0.021, *SE* = 0.009, *p* =.015. Namely, individuals who communicated their accomplishments more often, perceived more positive feedback, and in turn, were more optimistic in the following month. Finally, there was a marginally significant two-step indirect effect between capitalization attempts and positive emotions via perceived active-constructive feedback and optimism, b = 0.002, *SE* = 0.001, *p* =.057. This indicates that players who made more capitalization attempts tended to experience more positive emotions as a function of increased optimism from perceived more active-constructive feedback.

In contrast, players who perceived more active-destructive feedback were more likely to report a slight decline in self-esteem and an increase in gaming addiction. Lower self-esteem, in turn, was associated with increased gaming addiction symptoms. Consequently, there was a marginally significant indirect effect of active-destructive feedback on gaming addiction symptoms via decreased self-esteem, *b* = 0.009, *SE* = 0.005, *p*=.062. We also found a suppression effect. After controlling for the effects of active-constructive responses and self-esteem, optimism predicted a greater risk of gaming addiction despite predicting less gaming addiction in pairwise comparisons. This is a suppression effect because optimism predicted less risk of gaming addiction when studied in separation^88^. The potential indirect effect of capitalization attempts on gaming addiction via decreased self-esteem was marginally significant, *b* = −0.008, *SE* = 0.005, *p* =.066.

Demographic analyses indicated that younger participants were more likely to initiate capitalization attempts, *b* = − 0.195, *SE* = 0.046, *p* <.001. Younger participants also reported lower flourishing, *b* = 0.116, *SE* = 0.037, *p* =.002. The relationships between capitalization variables and psychological resources did not vary depending on the participant’s age, as indicated by non-significant interaction terms, all *p*s > 0.10.

In sum, in line with our hypotheses, capitalization attempts and perceived active-constructive feedback were associated with increases in psychological resources (self-esteem, self-efficacy, and optimism), and these increases were linked to enhanced positive emotions but not consistently to life satisfaction, negative emotions, and eudaimonic well-being. Mediation pathways through optimism and perceived active-constructive feedback were supported for positive emotions, but not for all proposed well-being outcomes. In contrast, perceived active-destructive feedback was related to decreases in self-esteem and increased gaming addiction symptoms. The hypothesized indirect effect via reduced self-esteem was marginally supported. Some expected pathways - such as indirect effects on life satisfaction and flourishing - did not reach significance.

## Discussion

Our findings showed that sharing accomplishments and perceiving active-constructive feedback from other players enhanced gaming-related psychological resources, which in turn improved daily emotional well-being. In contrast, players who perceived more active-destructive feedback experienced decreased self-esteem and were potentially more prone to gaming addiction symptoms. These results highlight the crucial role of positive peer interactions in cultivating a healthier and more supportive esports environment.

We observed that players who perceived active-constructive feedback developed a more optimistic outlook on future gaming experiences. This finding aligns with existing literature, highlighting the benefits of receiving responsive feedback^45^. Our findings demonstrated that active-constructive feedback is associated with greater psychological resources. Our study highlights links between negative communication—such as active-destructive feedback—and lower emotional well-being among players.

We found that players who perceived active-destructive feedback were at greater risk of experiencing self-esteem deterioration. As auto-regressive effects were controlled, we argue that the observed changes reflect state-like (month-to-month) fluctuations in self-esteem. These transient states were predicted by capitalization attempts and perceived active-destructive feedback from other players over the previous month. Such fluctuations introduce temporal variations beyond individuals’ stable, trait-like levels. The auto-regressive effects were comparable to those observed in previous studies^89^. Thus, our results underline the interplay between communicating accomplishments and perceived interpersonal feedback, highlighting their combined role in modifying players’ self-esteem beyond typical baseline levels.

In line with meta-analysis findings^43^, our results also indicated that players with lower self-esteem were more susceptible to gaming addiction. Therefore, the study highlights the potential for active-destructive feedback to contribute to gaming addiction, introducing a novel mechanism in the field. Non-enthusiastic capitalization was associated with feelings of inadequacy, which in turn were linked to lower self-esteem. This diminished self-esteem was associated with a higher likelihood of players engaging more intensively with games, possibly reflecting attempts to regain their image in the eyes of other players^90^. This underscores how negative social dynamics contribute to problematic gaming behaviors. This process adds to the more general link between lower self-esteem and gaming addiction, as lower self-esteem can result from many factors, including those outside of the gaming experience.

Moreover, we found that players benefited from more frequent capitalization interactions regardless of the type of feedback received. Specifically, individuals who more often shared their accomplishments with others experienced greater increases in self-esteem over the following month. This aligns with previous research^5,6,10^, highlighting the advantages of simply communicating positive events. However, careful consideration is needed to determine whether engaging other players by sharing one’s own accomplishments might outweigh the potential risk of receiving negative or active-destructive feedback.

We found a meaningful role of game-specific psychological resources in predicting well-being among players outside the gaming environment. Self-esteem led to changes in emotional well-being and gaming addiction symptomatology. Optimism was also essential, yet we did not find any benefits of self-efficacy. Whereas the findings regarding self-esteem and optimism support previous findings^34,35^, the limited role of self-efficacy contradicts previous studies^36^. Self-efficacy was related to well-being measures in pairwise comparisons. It was not significant after controlling for the autoregression of well-being measurements. This indicates that although self-efficacy and well-being tend to covary, higher self-efficacy is less related to explaining future changes in well-being.

Our findings regarding gaming optimism revealed mixed results. Initially, we observed a negative pairwise correlation between optimism and gaming addiction, indicating that players with higher optimism were less likely to exhibit symptoms of gaming addiction. This finding aligns with previous research highlighting optimism’s protective role among gamers. Optimistic individuals typically employ active, real-world coping strategies for stress, reducing their need to seek escapism through gaming. Additionally, these individuals often possess resilience and strong social resources, further protecting them from addictive behaviors^91^. However, subsequent multivariate analyses revealed suppression effects for gaming optimism^88^. When controlling for other positive predictors, optimism unexpectedly predicted higher gaming addiction symptomatology. Similar paradoxical associations involving optimism has also been documented in prior studies. Such discrepancies might stem from the complexity of optimism as a belief system, encompassing both adaptive and maladaptive (unrealistically positive) elements. Consequently, optimistic individuals may maintain functional positive beliefs while simultaneously holding dysfunctional beliefs. This interpretation is consistent with previous studies identifying positive outcome expectancies as predictors of internet addiction^92^. The underlying mechanism involves heightened positive reinforcement and expectancy effects enhanced by optimism, driving repetitive gaming behavior and dependency^93^. Since this unexpected finding was not initially hypothesized, further research is required to investigate gaming optimism’s nuanced and potentially dualistic structure.

Our results align with evidence that negative social interactions, such as toxic communication, prevalent in competitive gaming environments, can elevate stress and diminish player well-being^4,94^. For instance, active-destructive feedback mirrors the undermining effects of toxicity, which Hamari and Sjöblom identified as a barrier to enjoyment and engagement in esports^3^. Conversely, enthusiastic capitalization parallels effective team communication strategies that bolster morale and cohesion^95^. This suggests that active-constructive social feedback may serve as a buffer against the psychological strain of competitive gaming^96^.

In our multivariate model, we found support for several pathways linking interpersonal capitalization to psychological resources and well-being outcomes, although not all proposed relationships were significant. Specifically, negative emotions, life satisfaction, and flourishing showed no meaningful association with interpersonal capitalization. These findings partially support our broader theoretical model, emphasizing positive emotions and gaming addiction symptoms as key outcomes reflecting month-to-month interpersonal capitalization dynamics. The lack of association with negative emotions suggests that active-constructive feedback primarily enhances positive affect rather than directly reducing negative emotional states within esports contexts. This aligns with the theoretical conceptualization of interpersonal capitalization as predominantly boosting positive psychological states linked to success, rather than mitigating negative states^5,6^.

Finally, we found few differences between men and women, suggesting a possible narrowing of the gender gap in gaming. This is particularly noteworthy because previous research on interpersonal capitalization has indicated that women show greater engagement in facial expressiveness and physiological arousal during social interactions involving capitalization^9^. Additionally, younger participants were more likely to capitalize on their accomplishments, which may stem from a more substantial reliance on peer feedback among younger individuals^97^. However, older and younger participants exhibited similar associations between capitalization and psychological outcomes, as indicated by the non-significant moderation effect. This may suggest that although younger participants tend to capitalize on positive events more frequently, the psychological benefits derived from capitalization are comparable between younger and older participants.

### Limitations and future directions

Several limitations could be considered. First, our design warrants the generalizability of our findings to monthly intervals. This does not capture the full complexity of shorter or more prolonged interpersonal dynamics that would require the study of real-time gaming situations or much longer intervals spanning several months. Second, while the study’s design allowed the capture of changes and their precursors, direct manipulation of the independent variables might provide insights into the causality of the observed effects^82^. Third, our model did not include income and education as control variables due to large systematic differences across countries^98,99^. Future research examining demographic predictors of interpersonal capitalization in multi-country samples should explicitly account for these cross-country differences through more sophisticated modeling strategies, such as multilevel analyses.

Fourth, we measured perceived responses to capitalization attempts rather than actual feedback. Namely, we observed how the players who communicated their accomplishments perceived the feedback from other players rather than analyzing the feedback content directly. Previous experimental studies have validated the accuracy of such self-reports: individuals’ perceptions of feedback align with both partner-reported and independently observed responses^15,100^. However, future studies might use dyadic study designs to model capitalization processes in pairs of players directly because self-reports are prone to recall bias, social desirability, and individual differences in perception.

Fifth, the gaming time data collected in our study were unusable due to methodological challenges associated with the question. Although converging evidence suggests that gaming duration has minimal effects on players’ well-being^101–103^, further studies could control for this variable using more straightforward measures.

Sixth, our measure of perceived capitalization attempts did not differentiate between verbal and non-verbal communication channels, such as voice, text, gestures, or emoji^5^. Although prior experimental studies suggest correlations between verbal and non-verbal active-constructive responses^17,104^, future research should explicitly distinguish among communication modalities to evaluate their individual contributions to capitalization processes more precisely.

Seventh, we did not measure passive-constructive and passive-destructive social feedback due to constraints in survey length and to maintain a focused analysis. Previous studies indicate that only the active-constructive responses produce capitalization effects, while the passive or destructive responses produce adverse effects^105,106^. However, future research could incorporate these feedback types to comprehensively evaluate the social feedback processes.

Eighth, we used measures, e.g., for self-esteem, that capture more stable levels across a given period^89^. Studies focused on esports during organized events might prioritize explicitly measuring state self-esteem. Such measures might better reflect the psychological resources available on a given day, e.g., during a tournament^107^. Moreover, an in-depth exploration of self-esteem among players may benefit from distinguishing between contingent self-esteem (dependent upon successes in specific domains) and unconditional self-esteem, since pursuing contingent self-esteem, such as through achievements in esports, can incur substantial psychological costs^108^.

Ninth, we defined esports broadly to include both professional players participating in structured events with live audiences and hobbyist players engaging informally in competitive multiplayer gaming at home^1–3^. Nevertheless, some scholars adopt a narrower definition, limiting esports to competitive gaming during organized events^109^. Consequently, further research may increasingly focus on how interpersonal capitalization influences esports in more structured contexts.

Tenth, we observed that younger participants and those who made more capitalization attempts were more likely to discontinue the study after the first measurement. Although the dropout rate was relatively low and the differences were small-to-moderate for age and small for capitalization attempts, these results suggest that specific groups might be underrepresented in our longitudinal data relative to cross-sectional. Future research should include targeted strategies to reduce dropouts further, such as offering additional incentives. Additionally, future studies could incorporate qualitative measures, such as open-ended questions, to better understand the participants’ reasons for discontinuing after the initial measurement.

Finally, although our study involved a global population, it involved individuals fluent in English, which might not reflect broader cultural differences. Cultural norms around competition and social interaction could influence how feedback is given and received, potentially moderating the study’s findings.

### Practical implications

The study findings have implications for the esports community. First, they highlight the critical role of positive social interactions in enhancing players’ psychological resources and well-being, a relatively neglected area in gaming^4^. Encouraging positive feedback could foster a constructive environment, as positive responses are often reciprocated^9^. This could involve team-building communication training focused on constructive interactions. However, such feedback must be delivered honestly and based on actual performance. Overly positive evaluations, especially when inconsistent with recipients’ self-views, may conflict with self-verification motives and thus undermine trust and relational stability^110^.

Second, esports organizations could examine the benefits of community guidelines or peer-moderated forums that incentivize supportive dialogue, offering tangible rewards (e.g., in-game credits) for constructive engagement. These strategies could reward a supportive environment that enhances players’ well-being by bolstering resources like optimism and self-esteem and outcomes such as more positive emotions and less risk of gaming addiction. Thus, our findings underscore the potential of interpersonal capitalization to counteract the adverse effects of competitive stress and toxicity, aligning with prior calls for design-driven solutions to mitigate esports toxicity^4^. Future research could experimentally explore the efficacy of these regulations, testing their impact on player psychology and long-term engagement in esports.

Our results introduce a novel factor – active-destructive communication to accomplishments - considered in the context of gaming addiction. Invalidating the positive experiences of others through social feedback can damage players’ self-esteem and increase the risk of compensatory gaming preoccupation.

## Conclusions

Extending the concept of interpersonal capitalization to the gaming environment highlights the significant impact of computer-mediated social feedback on players’ psychological resources and the role of the resources in well-being. This research bridges a gap between traditional theories of interpersonal capitalization and the unique, socially dynamic context of esports that might inform future studies and interventions in the gaming community. We highlight the crucial role of positive peer interactions in fostering healthier psychological outcomes in the esports community.

## Supplementary Information

Below is the link to the electronic supplementary material.

Supplementary Material 1

Supplementary Material 2

Supplementary Material 3

## Data Availability

Data and analysis code are available at: https://osf.io/fr62k/.
